# Relation between fluid intelligence and mathematics and reading comprehension achievements: The moderating role of student teacher relationships and school bonding

**DOI:** 10.1371/journal.pone.0290677

**Published:** 2023-09-28

**Authors:** Cristina Semeraro, Pasquale Musso, Rosalinda Cassibba, Susanna Annese, Antonietta Scurani, Daniela Lucangeli, Alessandro Taurino, Gabrielle Coppola

**Affiliations:** 1 Department of Education, Psychology, Communication, University of Bari Aldo Moro, Bari, Italy; 2 Lower Secondary Public School «Michelangelo» Bari, Bari, Italy; 3 Department of Developmental Psychology and Socialization, University of Padova, Padova, Italy; Tallinn University: Tallinna Ulikool, ESTONIA

## Abstract

Several studies have shown the relevance among students of the quality of their interpersonal relationships for their academic achievement. Nevertheless, most studies available have explored the relation between the cognitive functioning and academic achievement without taking into account the quality of the relationships experienced in the school environment. Furthermore, the studies that have begun to consider the joint role of these factors in the prediction of academic achievement are scant. Therefore, it appears of relevance to deepen the relation between cognitive functioning and quality of school relationships in order to support students’ academic achievement and the potential of youth. In this paper, we examined the moderating role of the quality of student–teacher relationships and school bonding (STR-SB) in the associations of fluid intelligence (*Gf*) with academic achievement among adolescents (N = 219). A multiple-group structural equation modelling analysis revealed that STR-SB quality moderated unexpectedly only the link between *Gf* and mathematics. The findings support the idea that the quality of student–teacher relationships may be a relevant dimension to be considered to clarify the association between cognitive functioning and academic achievement.

## Introduction

An extensive body of research indicates that academic achievement may be a key aspect of adolescents’ life, as it is related to their later cognitive development, sense of competence and self-efficacy, as well as social and emotional well-being [e.g., 1–3]. Therefore, understanding how to catalyse mechanisms promoting academic achievement is critical not only for enhancing education programmes but also for fostering positive youth development.

In predicting school achievement, two distinct research lines can be traced: one line has stressed the role of intelligence [4–7] and a second line the role of interpersonal relationships [e.g., 8, 9]. In the former case, studies have focused attention on the role of fluid intelligence [3, 10, 11], which refers to the ability to adapt and deal with new situations in a flexible way, without previous learning being a decisive help. It is fundamentally shaped by primary skills, such as induction and deduction, relationships and classifications, the breadth of operational memory or intellectual speed [3, 12–16]. In the latter case, studies suggested that to succeed in school students need to develop positive (and at least non-negative) interpersonal relationships that may represent ecological resources, which might have a positive impact on school adjustment, engagement, and achievement [e.g., 17]. There is an extensive literature exploring the relation between the quality of student–teacher relationships and academic achievement [18–21]. Among others, the quality of student–teacher relationships and school bonding (STR-SB) has been found to be especially related to academic success [22, 23].

Even though these two research lines have worked substantially in an independent way, more recently there have been ongoing efforts to merge and comprehensively integrate the insights gained from each of them [24–29]. Some contributions have enlarged the focus from cognitive to non-cognitive individual factors [24, 27], as well as the complex interaction between them [30]. Recent contributions have expanded the focus beyond the individual level and have targeted factors allocated on the broader environmental level, such as the classroom, the relationship with the teachers, and the family context [30–32]. While confirming the strong association between cognitive measures and academic achievement, amply demonstrated by the scientific literature (i.e., between general domain measures and academic achievement), these recent contributions also suggest that non-cognitive measures (such as self-esteem, motivation, and quality of student–teacher relationship) could moderate such association [33–36]. In the same vein, our study intended to evaluate the moderating effect of a non-cognitive dimension (i.e., quality of student–teacher relationship and school bonding) on the association between fluid intelligence and academic achievement.

### Fluid intelligence and academic achievements

In a recent meta-analytic work, Peng et al. [3] investigated the relation between fluid intelligence (generally, labelled as *Gf*) and academic achievements, operationalised as mathematics and reading skills. They based this last choice on the most influential intelligence theories, i.e., Cattell and Horn’s fluid and crystallised intelligence model [37, 38] and Carroll’s [39] three-stratum model, as well as on the emphasis that mathematics and reading usually receive at school across various cultures. The findings revealed moderate but consistent reciprocal relation between *Gf* and mathematics and reading, with a stronger association with the former than with the latter. However, the authors also studied these relations as a function of various moderators, potentially explaining the variations evidenced in the literature. The relation between *Gf* and both mathematics and reading were moderated by distinct mathematics and reading skills, *Gf* tasks, and age. Specifically, *Gf* showed a stronger association with more complex mathematics (e.g., word problems) and reading (e.g., comprehension) skills than with foundational skills (e.g., calculation and code skills), *Gf* tasks of composite non-verbal reasoning had a stronger association with mathematics/reading compared to those of matrix and non-matrix reasoning, and all these *Gf* tasks showed stronger associations than visuospatial reasoning. Finally, the associations of *Gf* with mathematics/reading improved with age.

These findings were discussed in terms of different theoretical perspectives: (a) the intrinsic cognitive load theory [40], according to which the relation between *Gf* and academic performance is stronger when more complex tasks are considered; and (b) the mutualism theory [41], assuming weaker relation between *Gf* and mathematics/reading in early development but stronger associations in later development due to reciprocal influences. Although the results of Peng et al.’s work [3] provided an outstanding contribution to the current literature, the topic was especially focused on the links between cognition and learning. Other studies and other theoretical approaches suggest that for a more comprehensive understanding of the relation between *Gf* and academic achievements, it may be useful to also consider different and more social/ecological moderators.

### The potential moderating role of STR-SB quality in adolescence

The developmental systems perspective emphasises that human development inherently involves reciprocal influences between individuals and their changing contexts [e.g., 42]. With regard to youth, it proposes that when their strengths can be aligned with the resources of nurturing contexts, then these strengths may be optimised in positive developmental outcomes. This might be the case for students at school. Students have a number of potential strengths, such as their cognitive resources and specifically *Gf*. However, these resources may be actualised in academic achievement when the school context provides a positive environment in which the students’ strengths can be positively directed.

In line with this perspective, Vygotsky’s theory [43] claims that social influences are crucial to promote the potential of youth. Particularly, they develop their ability to independently perform and optimally use cognitive functions (as *Gf*) during their activity with significant adults in meaningful learning contexts, such as teachers at school. The consequences of these joint activities are usually related to academic achievement and higher school success [19, 44–47].

Although both these theories conceptualise an interaction between cognitive and relational domains, most studies have investigated them in a separate way. While past studies have mostly investigated schooling achievement at the level of the individual [e.g., 48–50], recent findings point to the importance of considering the role of factors allocated on a broader environmental level and related to students, parents, and teachers [19, 51, 52]. Recent studies have shown that the joint roles of individual and environmental factors are crucial to explain academic achievement [31, 32]. The relevance of considering the joint roles of these factors in schooling achievement (e.g., mathematics achievement) has also been highlighted in a recent review [30]. In their work, Chang and Beilock [30] showed how several studies provide converging evidence for individual (cognitive, affective/physiological, motivational) and environmental (social/contextual) factors that may explain the existence of a mathematics achievement gap between students. Individual factors (cognitive, emotional, social) interact with contextual ones in predicting schooling achievement.

Among these environmental factors, we decided to focus our attention on the quality of STR-SB in light of the relevance of this dimension in the schooling career of adolescents, as highlighted by recent reviews of the literature [17]. This line of study, rooted in attachment theory [53], proposes to conceptualise the relationship between teachers and students as an attachment relationship [54]. Similarly, as happens in relationships with caregivers, such attachment relationship will function as a secure base for the student to explore new learning opportunities as well as a safe haven in which to regulate negative emotions in the school context. Since this conceptualisation was shared in the academic community, it has been widely tested among preschool and primary school children that the affective quality of the student–teacher relationship is an important predictor of children’s academic achievement and schooling career, mediated by increased student engagement in the classroom setting [17, 45, 51, 55–57]. In contrast, difficult relationships with teachers and negative bonds with school have a negative impact on students’ motivation, their ability to adequately orient attentional resources, their positive participation in social learning activities, and, ultimately, their academic achievements [56, 58]. Particularly for children who are at risk of failure at school, an emotionally supportive relationship with a teacher can act as a protective factor and have positive effects on children’s developmental outcomes [45].

Instead, mixed and scant evidence exists for the role of STR-SB in adolescence. Some scholars argue that its importance declines over the course of an adolescent’s academic career, in part, due to changes in the social context: larger schools, less teacher–student interaction, and shifts in social support from teachers, peers, and parents [59]. Conversely, others have shown that STR-SB quality remains important for secondary school students’ achievement, and positive STR-SB is even more strongly associated with secondary school students’ achievement than with primary school students’ achievement [17]. A recent study examined STR-SB quality and students’ achievement in middle school and showed that positive STR-SB impacted mathematics achievement by reducing math anxiety [27]. To sum up, considering this state of the art, we suggest that this age deserves more research attention.

In the face of this scant empirical evidence, we might suggest that the influence of STR-SB changes from childhood to adolescence: later on, in fact, adolescents rely on teachers and school less than at earlier ages when they are primary relational resource. As such, instead of suggesting direct links between STR-SB and school achievement in adolescence, we might suggest that STR-SB quality becomes one important environmental variable, among others, moderating the essential links between the cognitive domain and school achievement [60]: high levels of warmth, closeness with teachers as well as a perceived positive bonds with school might support students to deploy effectively their cognitive resources to explore the learning environment, leading them to positive attitudes toward school and successful academic performance. Therefore, to contribute to the state of art of the literature, the current study focused on testing this latter possible mechanism among younger adolescents, based on an integration of intelligence theories, developmental systems perspective, and Vygotsky’s theory, as already illustrated.

### The present study

Starting from the state of the art of the above-mentioned literature, the present study aimed to test the moderating role of STR-SB quality on the relation between *Gf* and mathematics and reading comprehension achievements among young adolescents; that is, it focused on understanding how these relations strengthened or weakened depending on the STR-SB quality. Besides being a scantly investigated age in relation to the role played by STR-SB in school achievement, we chose this age group also because general cognitive abilities seem to be relatively more stable from this age onwards [24], despite more cognitive abilities being required in connection with new complex academic tasks [32]. In any case, it is relevant to highlight that in this period of life, physical development may not yet be complete [61]. In light of this and considering the possible repercussions on learning [62], measures of intelligence and of general cognitive functioning should be chosen in order to soften the impact of maturational variables. This choice would reduce the possible confounding effects on academic attainment due to fluctuations in the development of general cognitive abilities when testing for the moderating role of a third variable.

To achieve our goal, we adopted a person-centred approach [63]. Practically, this permitted to determine groups of students based on similarities in their STR-SB quality and to focus on how such groups may change for different students. The advantage in using such an approach is therefore to recognise the “tendency for a given person to have a distinct pattern of factors on which they are high, medium, or low” [64, p. 39], dealing simultaneously with non-linearity and interactions among variables that cannot be well described using variable-centred models [65]. Furthermore, this approach allows us to maximize the homogeneity within the groups and the heterogeneity between the groups, allowing us to grasp the effects due to the achievement of certain thresholds of certain characteristics. Starting from this, we investigated how the associations of *Gf* with mathematics and reading comprehension achievements changed according to these different groups. We were guided by the hypothesis that groups exhibiting higher levels of STR-SB quality showed stronger links of *Gf* with mathematics achievement and reading comprehension than groups exhibiting lower STR-SB quality. In fact, as previously mentioned, we deemed that the potentiality of cognitive resources, such as *Gf*, has a greater influence on academic achievement when student–teacher relationships and school context provide a (perceived) positive environment in which the students can maximise their strengths. In testing this hypothesis, we controlled for gender. Indeed, prior work has suggested gender differences in academic achievement, with male students showing better mathematics abilities and female students showing better verbal abilities than their respective counterparts [66, 67].

## Method

### Participants and procedure

Participants included a convenience sample of 219 sixth-grade students (54% male) from nine classrooms of one state middle school located in an urban area in southern Italy. This school serves a predominantly autochthonous community with a middle-high socioeconomic background, as emerged from the aggregate data provided by the same school about the index of family economic, social and cultural status (ESCS; see [67]): overall, the classes involved in this study presented ESCS = 0.98 (0 represents the average of the national reference population) and *SD* = 0.31 (expression of a limited variability). In Italy, the sixth grade represents the start of middle school, and this means that students have different teachers for different subjects; however, the teachers for the Italian language (including reading comprehension) and mathematics greatly represent the prevalent teachers with whom the most meaningful relationships are established. The sample excluded students with (a) clinical diagnoses of cognitive and leaning difficulties and (b) severe behavioural problems, as certified by mental health services. The mean age was 11.12 years (*SD* = .31). Ninety-seven percent of participants were Italian Caucasian, and all were Italian speakers.

All participants were informed of the objectives of the study and provided informed written consent before completing the investigation. The study had the prior approval of the Local Ethics Committee (code: CEL01/18) in accordance with the principles of the Declaration of Helsinki. The involved school was selected by using an internal departmental search database including a list of local school institutions. After selecting the schools attended by young adolescents, we sent a motivational letter of presentation of the study inviting them to participate. Finally, we selected the school that showed the best motivation to participate and, above all, to continue the research, given that the study had a longitudinal design (in the current study we reported data from the first wave). All sixth-grade classes and their students were included except for a minority (< 5%) of students who met the exclusion criteria (see just above). Participants’ parents were informed through schools about the purpose of this study and provided written informed consent for their children’s participation. For each school class, the data were collected in two collective, very close sessions during class time within the first school quarter. Participants had 1 hour in each session to complete the different tasks and/or questionnaires and could withdraw at any time.

### Measures

#### Fluid intelligence

Cattell’s Culture Fair Intelligence Test (CFIT; [68]) was used to assess fluid intelligence. The CFIT is a well-known matrix reasoning instrument assumed to be independent of cultural experiences. It includes two equivalent forms (A and B). As suggested by the test manual, form A is preferable in a school setting [see, 68], and it is characterised by four subtests involving multiple-choice problems progressing in difficulty: series (12 items), classification (14 items), matrices (12 items), and conditions (8 items). A raw score for each subtest is calculated by summing the correct responses and the total raw score ranging from 0 to 46. The validity of the CFIT has long been established [24, 69], and the subtest raw scores are usually taken to be strong indicators of one comprehensive latent construct of fluid intelligence; this is why in the present study we did not consider the norms reported in the manual, which have a greater utility in clinical assessment. The Spearman-Brown split-half reliability coefficient in this study was .79 for the entire measure.

#### Mathematics achievement

The AC-MT 11–14 standardised mathematical battery [70] was used to assess mathematics achievement. This test was developed for the assessment of calculation, arithmetic reasoning, and number comprehension skills of Italian students attending middle schools. The AC-MT 11–14 tasks can be grouped into three areas: written calculation (8 items), referring to procedural aspects of mathematics (i.e. written operations, such as addition, subtraction, etc.), number knowledge (20 items), referring to the aspects of estimating the quantity and positional values of numbers (identify the largest number, transform into written digits, etc.), and mathematic reasoning (32 items), referring to mental arithmetic task (approximate calculation, mathematical facts, rounding tests, etc.).These three constructs were assessed with increasingly complex test questions. A raw score for each task is calculated by summing the correct responses, and the total raw score range is 0–60. The battery has demonstrated good internal consistency and validity [71, 72]. The Spearman-Brown split-half reliability coefficient in this study was .76 for the entire measure.

#### Reading comprehension

The standardised MT battery [73] was used to assess reading comprehension of Italian students attending middle schools. This test was developed to assess the ability to gather correct information from the reading of a text independently of the contribution of decoding and memory processes. Specifically, it explores the ability to make inferences, lexical competence, vocabulary knowledge, strategic processing, and metacognition. Participants are asked to read two passages of approximately 25 lines, one of a narrative type and one of an informative type, and then to answer 15 questions for each passage about what they had read. A raw score for each task is calculated by summing the correct responses, and the total raw score ranges from 0 to 30. The battery has demonstrated good internal consistency and validity [74, 75]. The Spearman-Brown split-half reliability coefficient in this study was .70 for the entire measure.

#### The quality of student–teacher relationships and school bonding

The Italian adaptation of the Student–Teacher Relationship and School Bonding Questionnaire (STR-SB_Q; [23, 76]) was used to assess students’ self-reported perceptions of relationships with Italian language and mathematics subject teachers and bonds with school. Specifically, STR-SB_Q examines the perceived positive and negative—both affective and cognitive—experiences of warmth, trust, accessibility, and responsiveness of student–teacher relationships, along with the general perceptions of the overall school environment. The questionnaire is composed of 19 self-assessment items, which can be grouped into 3 subscales: affiliation with teacher (eight items; e.g., “I trust my teacher”), dissatisfaction with teacher (three items; e.g., “I feel angry at my teacher”), and bonds with school (eight items; e.g., “I feel safe at school”). These constructs are also good proxy variables with reference to the relational dimensions postulated by extended attachment theory. Affiliation with teacher is close to the “closeness” construct, while dissatisfaction with teacher is an indirect indicator of a climate of potential “conflict”. Bonds with school extends the concept of closeness to the whole school environment [54]. Items were rated on a 4-point Likert-type scale, from *never or almost never true* (1) to *almost always or always true* (4). In the current study, Cronbach’s alphas were .88, .66, .80 for affiliation with teacher, dissatisfaction with teacher, and bonds with school, respectively. Notwithstanding the values for dissatisfaction with teacher could appear low (< .70), it evidenced a sufficient level of internal consistency considering that this subscale comprises only three items (Cronbach’s alpha is highly sensitive to the number of items). Furthermore, the average item-total correlations were .48, which was higher than the acceptable level of .30 suggested by Nunnally and Bernstein [77], indicating that the different groups of items were measuring the construct in the same direction.

### Data analysis

Descriptive statistics for the observed variables were initially calculated. Afterward, as also suggested by Murray and Greenberg [23], we conducted a cluster analysis based on the STR-SB_Q subscale scores to identify groups of students relative to their perceptions of their STR-SB profiles. We identified the appropriate number of clusters by hierarchical cluster analysis, using Ward’s method based on the squared Euclidean distance [78] and examining solutions from two to four clusters. The a priori criteria used for choosing the final number included the theoretical meaningfulness of each cluster, parsimony, and explanatory power (see [79]; the cluster solution had to explain at least 26% of the variance in each of the STR-SB_Q dimensions; see [80]). Sequentially, we grouped the participants by *K*-means cluster analysis procedures, and standardised mean values of the STR-SB_Q grouping variables were illustrated. The validity of the final solution was checked via multivariate analysis of variance (MANOVA) on the three STR-SB_Q dimensions by cluster. We also tested the replicability of the solution by splitting the sample into two random halves and recomputing the cluster analyses for each subsample. Level of agreement was calculated using Cohen’s [81] kappa.

After reporting bivariate correlations among the observed variables of interest for each STR-SB profile group, we took a multigroup structural equation modelling (SEM) approach to test our hypotheses using *Mplus 7* [82]. First, we performed a confirmatory factor analysis (CFA) on the entire sample to test a measurement model including the latent variables of *Gf* (series, classification, matrices, and conditions as indicators), mathematics achievement (MA; written calculation, number knowledge, and mathematic reasoning as indicators), and reading comprehension (RC; scores for each of the two passages to be read as indicators). We permitted each indicator’s factor loading on the hypothesized factor to be freely estimated while fixing at zero the cross-loadings. Factor covariances were allowed. To examine measurement invariance across the STR-SB profile groups, we conducted multigroup CFAs, sequentially introducing appropriate constraints to test different levels of invariance: equal factor structure constraints for configural invariance and equal factor loading constraints for metric invariance [83]. Second, we estimated a multigroup SEM (M1) so that the pathways from *Gf* to MA and RC were freely estimated across the STR-SB profile groups. Gender (dummy coded: 0 = female; 1 = male) was controlled by allowing it to predict both MA and RC. M1 was compared with two other more restrictive models, one (M2) constraining the pathways from *Gf* to MA to be equal across profiles and the other (M3) constraining the pathways from *Gf* to RC.

We evaluated the model fit according to the most popular and widely employed fit indices and their best associated cut-offs [84]: the chi-square (χ^2^) with *p*-value > .05, CFI ≥ .95, and RMSEA ≤ .06. Significant differences among nested models (the more restrictive vs less restrictive) were evaluated by using of the following criteria: χ^2^ difference (Δχ^2^) significant at *p* < .05 and ΔCFI < -.005 [85].

## Results

### Preliminary analyses

Only a few missing values were found for the study variables (3%). We performed missingness analyses to explore if participants with missing data were systematically different from participants with complete data. Results supported the missing completely at random assumptions and, therefore, missing values were imputed at item level using a regression estimation function. Table 1 summarizes the descriptive statistics and shows how some observed variables were not normally distributed with skewness and kurtosis values >|1.00| [84]. As multivariate non-normality was also evidenced (normalized Mardia’s coefficient was 7.82, *p* < .001), the data were subsequently analysed using robust maximum-likelihood estimation methods in the context of SEM models. Bivariate correlations among STR-SB variables were: (a) *r* = -.60 between affiliation with teacher and dissatisfaction with teacher, (b) *r* = .64 between affiliation with teacher and bonds with school, and (c) *r* = -.43 between dissatisfaction with teacher and bonds with school.

**Table 1:** Means, standard deviations, skewness, kurtosis, and minimum/maximum values of standardized scores for the key study variables for the entire sample and the STR-SB quality group.

		*M*	*SD*	Skewness	Kurtosis	Min. stand.	Max. stand.
Entire sample (*N* = 219)							
1.	CFIT—series	8.63	1.82	-1.38	3.82	-4.74	1.85
2.	CFIT—classification	7.05	1.89	0.21	0.09	-3.19	3.14
3.	CFIT—matrices	8.33	2.12	-0.60	0.10	-3.46	1.73
4.	CFIT—conditions	3.83	1.81	-0.12	-0.80	-2.12	2.31
5.	MA—written calculation	6.07	1.61	-0.72	-0.13	-3.05	1.16
6.	MA—number knowledge	16.16	3.40	-1.65	3.49	-4.57	1.09
7.	MA—mathematic reasoning	19.25	4.92	-2.07	4.73	-3.77	0.93
8.	RC—comprehension_1	8.79	2.50	-0.11	-0.23	-2.63	2.41
9.	RC—comprehension_2	9.53	2.57	-0.55	0.67	-3.59	2.06
10.	STR—affiliation with teacher	25.60	3.94	-0.78	0.41	-3.41	1.59
11.	STR—dissatisfaction with teacher	6.50	2.24	0.85	0.22	-1.08	3.26
12	STR—bonds with school	17.88	3.08	-0.47	-0.02	-2.82	1.94
High STR-SB quality group (*n* = 119)							
1.	CFIT—series	8.71	1.82	-1.22	8.71	-4.19	1.85
2.	CFIT—classification	7.12	2.03	0.09	7.12	-3.19	3.14
3.	CFIT—matrices	8.58	2.00	-0.35	8.58	-2.04	1.73
4.	CFIT—conditions	3.78	1.72	-0.07	3.78	-2.12	2.31
5.	MA—written calculation	6.20	1.62	-0.83	6.20	-2.45	1.16
6.	MA—number knowledge	16.18	3.76	-1.90	16.18	-4.57	1.09
7.	MA—mathematic reasoning	19.66	5.04	-2.39	19.66	-3.77	0.93
8.	RC—comprehension_1	9.15	2.34	-0.54	9.15	-2.63	2.02
9.	RC—comprehension_2	9.73	2.32	-0.76	9.73	-3.59	2.06
10.	STR—affiliation with teacher	28.37	1.86	-0.52	28.37	-1.16	1.59
11.	STR—dissatisfaction with teacher	5.51	1.54	0.99	5.51	-1.08	1.52
12	STR—bonds with school	19.49	2.29	-0.75	19.49	-1.87	1.94
Low STR-SB quality group (*n* = 100)							
1.	CFIT—series	8.52	1.83	-1.61	4.84	-4.74	1.30
2.	CFIT—classification	6.96	1.72	0.39	-0.43	-1.61	2.09
3.	CFIT—matrices	8.03	2.22	-0.77	0.50	-3.46	1.73
4.	CFIT—conditions	3.88	1.91	-0.19	-1.10	-2.12	1.76
5.	MA—written calculation	5.93	1.60	-0.62	0.10	-3.05	1.16
6.	MA—number knowledge	16.14	2.94	-0.94	0.53	-2.31	1.09
7.	MA—mathematic reasoning	18.77	4.76	-1.73	3.77	-3.77	0.93
8.	RC—comprehension_1	8.36	2.62	0.36	-0.15	-2.24	2.41
9.	RC—comprehension_2	9.30	2.82	-0.33	-0.27	-2.84	2.06
10.	STR—affiliation with teacher	22.32	3.16	-0.80	1.33	-3.41	1.59
11.	STR—dissatisfaction with teacher	7.68	2.38	0.35	-0.36	-1.08	3.26
12	STR—bonds with school	15.97	2.79	-0.14	0.28	-2.82	1.94

*Note*. STR-SB = student-teacher relationships and school bonding; Min. stand. = Minimum value of standardized score; Max. stand. = Maximum value of standardized score.

The principal descriptive statistics and the correlation matrix are reported in Table 1.

### Cluster analysis

**Fig 1:**
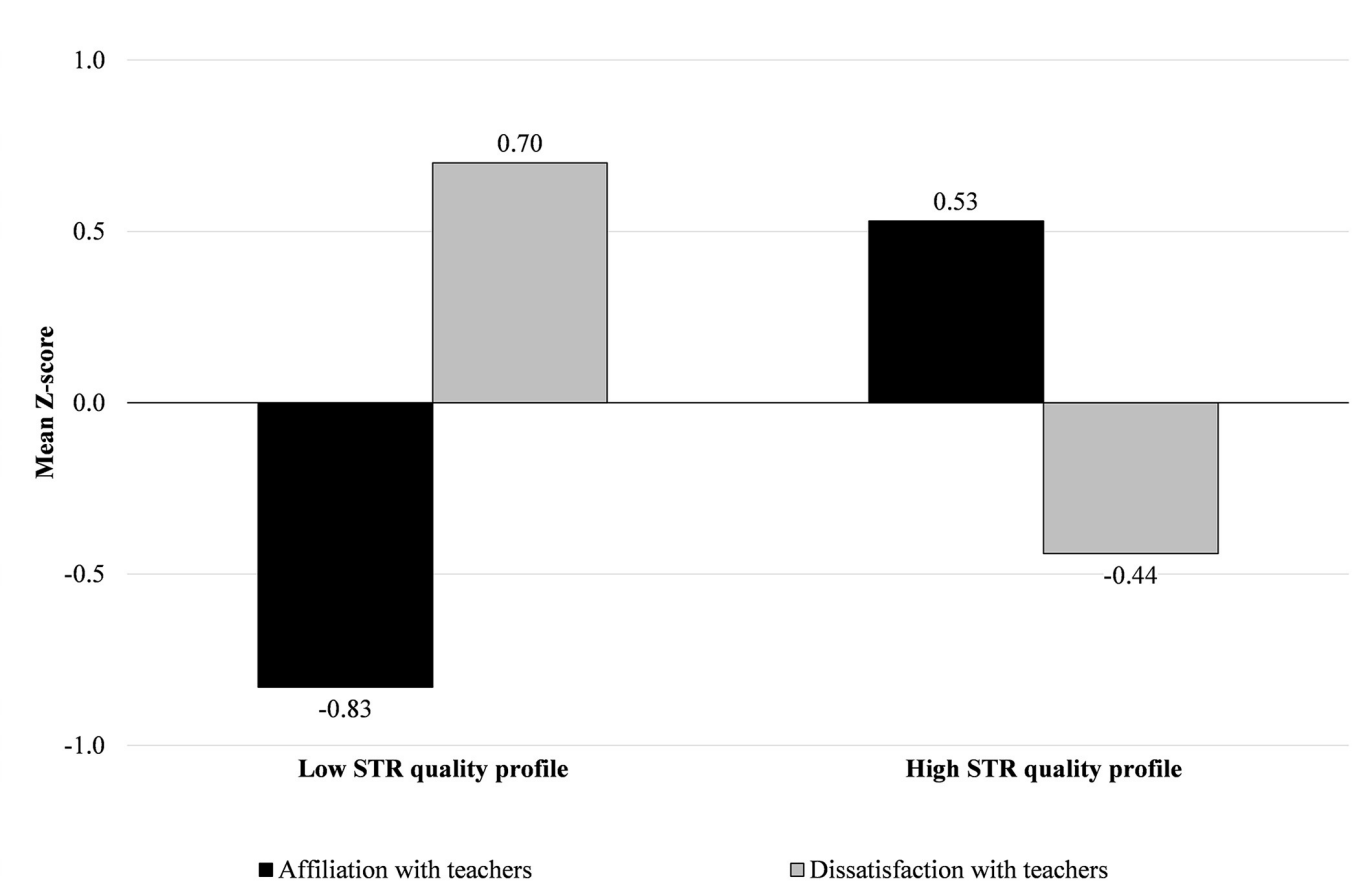
Mean Z-scores for affiliation with teacher, dissatisfaction with teacher, and bonds with school by the two STR quality profiles. STR = student-teacher relationships and school bonding.

Based on the a priori criteria, a two-cluster solution was retained as the most appropriate. Solutions with a greater number of clusters violated the principles of parsimony, explanatory power, and/or theoretical meaningfulness [79], including clusters that presented slight differences compared to the two clusters and that were scarcely interpretable. The first cluster (*n* = 100; 46% of the sample) consisted of students scoring higher on dissatisfaction with teacher and lower on affiliation with teacher and bonds with school. The second cluster (*n* = 119; 54% of the sample) was composed of adolescents who scored higher on affiliation with teacher and bonds with school and lower on dissatisfaction with teacher. Thus, we found, in sequence, groups with low and high perceived STR-SB quality (see Fig 1 for standardised means and Table 1 for descriptive statistics). Furthermore, the MANOVA of the grouping variables revealed a significant multivariate effect, Wilks’ Lambda = .40, F (3, 215) = 106.90, *p* < .001, η^2^ = .60, indicating that about 60% of the variability was accounted for by group differences between the two clusters. Also, subsequent univariate analyses of variance revealed that the two-cluster solution explained a good percentage of variance for each grouping variable (about 40% on average). Findings from the replicability analysis revealed a *k* = .83, indicating a good level of reliability and stability.

### Multiple-group SEM analysis

**Fig 2:**
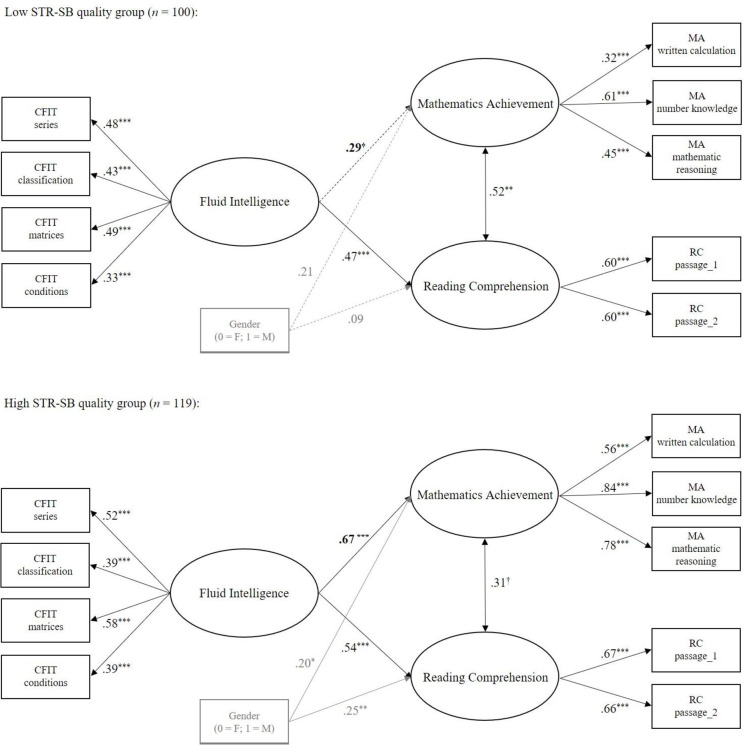
Final estimated multiple-group model illustrating the moderating effect of the STR quality in the link of fluid intelligence with mathematics achievement (but not with reading comprehension). STR = student-teacher relationships. CFIT = Cattell’s Culture Fair Intelligence Test. MA = Mathematics Achievement. RC = Reading Comprehension. *Note*. Standardized coefficients are shown. Solid lines represent significant, and dashed lines nonsignificant, pathways at *p* < .05. Coefficients in bold are significantly different across groups. Gender, as controlling variable, and related pathways are represented in light grey. Residuals are not shown for brevity. ^†^*p* < .10, *p < .05 *.

Correlations between the main and control (gender) indicator variables, as well as between the main latent variables, by STR-SB profile group are displayed in Table 2. The measurement model fit the data well, χ^2^(24) = 28.53, *p* = .26, CFI = .980, RMSEA = .029, and fully metric measurement invariance across STR-SB profile groups was evidenced (see Table 3). Starting from the metric invariant model, we ran the model M1 (pathways from *Gf* to MA and RC free to be estimated), showing good fit, χ^2^(74) = 81.42, *p* = .26, CFI = .971, RMSEA = .030. When comparing it with the more restrictive M2 (pathways from *Gf* to MA constrained to be equal), we obtained a significantly worse fit for M2, Δχ^2^(1) = 6.00, *p* = .01, ΔCFI = —.020. There was no significant change in the model fit when comparing M1 and M3 (pathways from *Gf* to RC constrained to be equal), Δχ^2^(1) = 0.12, *p* = .73, ΔCFI = .004. This suggests that the association between *Gf* and MA, but not between *Gf* and RC, was moderated by the STR-SB quality profile as shown in Fig 2, representing the final estimated model, M3. In particular, the positive relation between *Gf* and MA was significantly stronger in nature for the high STR-SB quality group than for the low group. Instead, the positive association between *Gf* and RC was not significantly different between the two profiles groups.

**Table 2:** Correlations for key indicator and latent study variables, by STR-SB quality group.

		1.	2.	3.	4.	5.		6.		7.		8.		9.		10.
Indicator variables																
1.	CFIT—series		.27**	.23*	.18	.19*		.31***		.28**		.22*		.29**		.01
2.	CFIT—classification	.17		.28**	.23*		.09		.16		.15		.07		.19*	-.01
3.	CFIT—matrices	.21*	.23*		.19*	.24***		.30***		.36***		.27***		.23*		.02
4.	CFIT—conditions	.23*	.12	.23*		.25**		.27**		.24**		.06		.01		.18*
5.	MA—written calculation	.08	-.02	-.08	.06			.44***		.48***		.25**		.17		.06
6.	MA—number knowledge	.07	.05	.12	.09	.23*				.65***		.42***		.35***		.22*
7.	MA—mathematic reasoning	-.08	.08	.14	.26**	.20*		.23*				.25**		.25**		.17
8.	RC—comprehension_1	.03	-.03	.05	.10	.00		.22*		.08				.45***		.27**
9.	RC—comprehension_2	.30**	.21*	.13	.11	.25*		.24*		.13		.36***				.08
10.	Gender (0 = F; 1 = M)	.02	.11	.05	-.02	-.11		.23*		.06		.08		.05		
Latent variables																
1.	CFIT		.68***	.57***												
2.	MA	.21		.60***												
3.	RC	.39	.31													

*Note*. Lower diagonal: correlation matrices for data related to students in the low STR-SB quality group (*n* = 100). Upper diagonal: correlation matrices for data related to students in the high STR-SB quality group (*n* = 119). STR-SB = student-teacher relationships and school bonding. CFIT = Cattell’s Culture Fair Intelligence Test. MA = Mathematics Achievement. RC = Reading Comprehension.

*p < .05

**p < .01

***p < .001.

**Table 3:** Multi-group CFA goodness-of-fit indices for the measurement model including the latent variable of Fluid Intelligence (Gf), mathematics achievement, and reading comprehension across the STR-SB quality groups.

Model	χ^2^ (df)	*p*	CFI	RMSEA	Δχ^2^ (Δ*df*)	*p*	ΔCFI
Equal factor structure (configural invariance)	55.61 (54)	.41	.993	.016	-	-	-
Equal factor loadings (metric invariance)	62.63 (60)	.38	.989	.020	7.00 (6)	.32	-.004

*Note*. CFA = confirmatory factor analysis. STR-SB = student-teacher relationships and school bonding.

## Discussion

The present study investigated the role played by the relationship of students with teachers and schools in promoting academic achievement. Specifically, our aim was to test whether STR-SB moderated the relation between *Gf* and mathematics achievement and reading comprehension among younger adolescent students. We hypothesised that students with higher STR-SB quality showed stronger links between *Gf* and mathematics/reading than students with lower quality.

Generally, we found significant or close-to-significant associations of *Gf* with mathematics achievement and reading comprehension after controlling for gender. This result is consistent with previous studies documenting significant positive relations between *Gf* and academic attainment [3]. In our sample, on average, such association was moderate in magnitude, suggesting that *Gf* is operationally distinct from mathematics/reading achievement, in accordance with the Cattell-Horn-Carrol theory [37–39], and a useful conceptual tool for understanding academic achievement processes [86]. Thus, during the first year of middle school, when novel situations and academic tasks of asked of younger adolescent students, the deliberate use of *Gf* mental operations (e.g., making inferences or classifications, producing and testing hypotheses, and solving problems; [87]) may be an important correlate of academic performance.

However, our study went beyond previous research, showing that the relation between *Gf* and academic success may be moderated by STR-SB quality, depending on a domain-specific achievement perspective. Our data showed a stronger link between *Gf* and mathematics achievement but not with reading comprehension in the high-STR-SB-quality group compared to the low one. Thus, our moderation hypothesis was only partially supported.

We suggest that one mechanism in action explaining why a high student-teacher relationship enhances the relation between *Gf* and school achievement might be the so-called principle of investment [37], according to which the degree of investment of fluid intelligence depends also on the variety of opportunities and programs affordable in the learning environment, which promotes the acquisition of knowledge and skills, and the development of academic achievement. With this respect, we suggest that students experiencing intimate relationships with the teachers and feeling closely bonded to the school, might not only have increased opportunities to learn but might use such opportunities more effectively, thanks to their motivation, self-regulation and ability to regulate themselves at a metacognitive level during the learning process [54, 88, 89]. As such, they invest *Gf* more effectively compared to their counterpart with low STR-SB, leading to higher school achievement.

More in general, the moderation effect we found may also be interpreted as the result of a successful or failed alignment process [42] between students’ cognitive resources (e.g., *Gf*) and STR-SB quality. As suggested by Vandenbroucke et al. [90] in a recent meta-analytic work including 2- to 12-year-old children, for students involved in positive interactions and bonds with teachers and school, this alignment mechanism may promote an increased exploratory and engagement capacity. Such students seem to feel more self-confident and safer in their environment and are able to face and persist even when faced with somewhat difficult tasks. These stimuli and experiences represent challenges that favour the formation of a new and more complex use of cognitive abilities in the school context. From this point of view, also, and perhaps particularly, *Gf* could develop better and more as a result of these stimulating activities, with consequences also on school attainment. However, the authors also suggested a second potential mechanism related to lower levels of stress when relationships and bonds with teachers and school are positive, as shown by lower salivary cortisol levels in students. This would allow to keep the stress levels in the average range (levels that are too high or too low are usually worse conditions), ensuring adequate cognitive and academic performance. Our findings are in line with both these two explanations and allow for extending them up to early adolescence. In addition, both Blair [91] and Pekrun et al. [92] posited that negative emotions, like anger, reduce achievement partly because they negatively affect higher-order cognitive processes (such as problem-solving, memory, and strategic thinking) and focus attention on a narrow set of behavioural options [93]. There is substantial evidence that cognitive processes are strongly related to achievement; thus, evidence that negative emotions probably experienced in low-quality relationships are linked to these processes is consistent with the notion of moderation. In fact, relationship-related anxiety and anger may disrupt both students’ ability to recall relevant material and academic success [94–97; for a meta-analysis, see 98]. As Blair [91] noted, young individuals characterised by negative emotionality are likely to have a hard time applying higher-order cognitive processes simply because their emotional responses do not call for reflective planning and problem-solving, so these skills are underused and underdeveloped. When a student’s experience of relationship-related negative emotion leads to focusing on the object of the emotion (as when a child ruminates on the morning’s event that resulted in their anger), cognitive resources are diverted away from educational materials to events or circumstances that distract from learning. In this way, low-quality relationships resulting in negative emotions could interfere with scholastic activities by reducing resources needed to integrate and recall important details. This seems particularly valid for mathematics tasks that usually need more cognitive resources because they are mostly linked to the abstract domain and furthermore are not as common in everyday life as verbal comprehension and reading tasks.

Nevertheless, this process seems to be dependent on the specific domain of achievement; namely, it seems to characterise mathematics achievement but not reading comprehension achievement. In formulating a possible interpretation of this result, we considered the relevance of non-cognitive variables, such as the emotional-motivational ones widely discussed in the literature in reference to academic achievement in mathematics. In scientific subjects, non-cognitive measures could play an important role in performance by interfering or facilitating the deployment of more domain-general cognitive resources, such as attentional resources, working memory, or efficiency of executive functions [99, 100]. These aspects certainly require further study, as the relations between these constructs are extremely complex. Another possible explanation is that individuals are not only more exposed to reading from childhood than mathematics [101], but they also receive more support for learning to read outside of school, for example through the explanations given by parents or grandparents in everyday situations [19, 51, 52]. In line with Vygotsky’s theory [43], these social influences may be equally important to promote the potential of youth compared to those provided by teachers at school. Thus, it might be that students at middle school developed or are developing their ability to use cognitive functions (as *Gf*) in the process of reading comprehension during activities with different significant adults. This could dampen the impact of the moderating effect of STR-SB quality on the association between *Gf* and reading comprehension. A further plausible explanation is linked to the specific reference context of the research. Historically, Italy is a country where much relevance has been given to verbal-linguistic rather than logical mathematical subjects, with the consequence that the idea that mathematics is a difficult and complicated discipline to learn is widespread [102]. In this context, a positive STR-SB might have a buffering effect by helping students to be more aware that they can make the most of their resources even when studying mathematics [103]. Lastly, we cannot exclude that methodological choices might have impacted the differential moderating effect of STR-SB. The result could in fact depend on the task selected for the evaluation of comprehension which is an ability that has been shown to rely more on non-language-specific abilities [104], and, therefore, might be scarcely susceptible to be influenced by teacher’s intervention. Probably the selection of a single word reading task or reading text task could be more linked to a direct intervention of the teacher, as well as being linked to phonological skills which are usually a target of the teacher’s intervention [105].

### Limitations and implications

There are several limitations to be noted when interpreting our results. First, our sample came from a single school because of the need to prospectively follow the participants longitudinally in the simplest and most precise way. Moreover, its size was quite limited, and it was ethnically homogenous. This might limit the generalizability of the results. Second, we assessed the quality of the relationships only by asking students to consider the Italian language and mathematics subject teachers. However, these general evaluations of relationships with teachers may be biased because the subject (liked or not, for example) may influence the students’ assessment of the teachers. Also, there are many studies describing how teacher preference may influence, and be influenced by, students’ relationships. Further investigations should take this into account to better clarified this potential confounding factor. Third, we did not assess the teachers’ perspectives of the STR-SB quality due to limits imposed by the times and commitments provided by the school. Indeed, it would be very useful to have multi-perspective information on STR-SB quality to better understand how relational factors can influence both general cognitive resources and academic performance [106]. Fourth, we focused on just one possible cognitive correlate, namely *Gf*, and one possible non-cognitive correlate, namely STR-SB, of academic achievement. These are certainly crucial constructs to be considered, but additional factors deserve consideration, including, for example, evaluation of the quality of relationships with teachers of specific subjects (and not with teachers in general). Fifth, the cross-sectional nature of this study precludes us from clearly drawing conclusions about the direction or the reciprocity [3] of the associations between *Gf* and mathematics/reading achievements as well as the stability of the moderating role of STR-SB quality. In line with this, other alternative models to be compared to our proposed model might be hypothesized, for example one whereby academic achievement affects relationships within the school [54, 107]. Longitudinal data could draw clearer conclusions about both the associations between the studied variables and the developmental processes involved.

Despite these shortcomings, our findings have relevant implications. They indicate the importance of student–teacher relationships and bonds with schools relative to specific domains of academic achievement (i.e., mathematics), which, in turn, might be associated with more general positive youth development outside of school [60, 103]. This encourages the creation and implementation of interventions aimed at supporting high STR-SB quality to stimulate school success. In this line, for example, a number of recent teacher professional development programmes have shown a positive impact on STR-SB [108, 109]. Moreover, our results could help explain and improve the mixed outcomes of cognitive training, with most studies reporting no effects from training on *Gf* or academic achievements [110, 111]. One explanation is that the relationship between trainers and students is a fundamental dimension for training to be effective. Ensuring that this relationship is qualitatively positive may lead to conditions for a better expression of students’ personal resources, both in terms of general cognitive processes and academic performance. Furthermore, another implication is that schools should strive to improve the socio-emotional climate of classes, and to do so, they could systematically introduce measures of the STR-SB from multiple points of view. This could favour an evaluation of the relational processes within the class, the implementation of corrective interventions when the relationships are on the negative side, and, in the long term, an improvement in school performance among students.
